# Proteomic Changes Associated With Endogenous *FBXW7* Mutations in Moderately Differentiated Endometrial Cancer Cells Include Increased TROP2 and Galectin‐3 Levels

**DOI:** 10.1002/cam4.70765

**Published:** 2025-03-14

**Authors:** Mary Ellen Urick, Suresh Kumar Chalapareddy, Eun‐Jeong Yu, Daphne W. Bell

**Affiliations:** ^1^ Reproductive Cancer Genetics Section, Cancer Genetics and Comparative Genomics Branch National Human Genome Research Institute, National Institutes of Health Bethesda Maryland USA

**Keywords:** endometrial neoplasms, FBXW7, Galectin‐3, mutation, proteome, TROP2

## Abstract

**Background:**

Endometrial cancer (EC) is the fourth most commonly diagnosed cancer among women in the US and the fifth leading cause of cancer death in this population. The *FBXW7* tumor suppressor gene is frequently mutated in all molecular subtypes of EC. The encoded protein is part of a ubiquitin ligase complex that targets substrate proteins for ubiquitination and, in most instances, proteasome‐mediated degradation.

**Aims:**

The purpose of this investigation was to identify the proteomic changes associated with endogenous *FBXW7* mutations in EC.

**Materials & Methods:**

Quantitative LC–MS/MS was used to identify significant (*p* < 0.05) differences in the proteomes and phosphoproteomes of two *FBXW7*‐mutated EC cell lines, HEC‐1‐B^FBXW7−R367X^ and JHUEM‐1^FBXW7−R505C^, as compared to isogenic mutation‐corrected cell lines. Western blotting was performed to orthogonally validate a subset of protein changes.

**Results:**

Analysis of LC–MS/MS results identified 397 total proteins and/or phosphoproteins with significantly different levels in both HEC‐1‐B^FBXW7−R367X^ and JHUEM‐1^FBXW7−R505C^, as compared to isogenic mutation‐corrected cell lines. This protein set included increased levels of TROP2, galectin‐3, ASS1, and PLCG2 in both HEC‐1‐B^FBXW7−R367X^ and JHUEM‐1^FBXW7−R505C^ cells; these perturbations orthogonally validated by western blotting.

**Conclusion:**

This study provides novel insights into the proteomic and phosphoproteomic effects of the endogenous FBXW7^−R367X^ and FBXW7^−R505C^ mutations in EC cells, including increased levels of galectin‐3, a potentially druggable target, and of TROP2, which is a druggable target in EC.

## 1 Introduction

Endometrial cancer (EC) is currently the fifth leading cause of cancer death among women in the United States (US) [1], and by 2040 it is expected to become the 4th leading cause of cancer death for US women [2]. Endometrioid ECs (EECs) are the most frequently diagnosed histological subtype, representing approximately 80% of new diagnoses and causing approximately 50% of EC deaths. The majority of non‐endometrioid tumors are serous, clear cell, or metaplastic ECs (uterine carcinosarcomas). Endometrioid and serous ECs segregate across four prognostically distinct molecular subgroups in The Cancer Genome Atlas (TCGA): copy number high/serous‐like, microsatellite instability (MSI)/hypermutated, copy number low/microsatellite stable (MSS), and POLE/ultramutated ([3]; reviewed in [4]). The POLE/ultramutated subgroup had the best prognosis, and the copy number high/serous‐like subgroup had the worst prognosis [3].

The FBXW7 tumor suppressor protein is a subunit of a ubiquitin ligase complex that regulates proteolytic and non‐proteolytic ubiquitination of substrate proteins [5, 6]. The functional domains of FBXW7 include a C‐terminal WD repeat region that mediates FBXW7‐substrate binding. *FBXW7* is one of the most frequently mutated genes in EC (reviewed in [4]). Somatic mutations in *FBXW7* occur in 10%–12% of endometrioid, 15%–29% of serous, 10%–39% of clear cell, and 13%–25% of metaplastic ECs ([3, 7]; and reviewed in [4]). The R465, R479, and R505 residues within the WD repeats mediate binding of FBXW7 to proteins targeted for ubiquitination (reviewed in [5]), and are mutation hotspots in EC and in some other tumor types [8]. Studies in other cancer cell types have shown that FBXW7 hotspot mutants at residues R465 and R479 are dominant‐negative mutants that fail to bind substrate proteins, resulting in elevated substrate levels [8].

Despite the frequent occurrence of *FBXW7* mutations in EC and the pathogenicity of *Fbxw7* deletion in a genetically engineered mouse model of uterine cancer [9], little is known regarding the functional consequences of *FBXW7* mutations in the context of human EC cells. Because many FBXW7 substrates are oncoproteins that regulate gene transcription or signal transduction, we hypothesized that *FBXW7* mutations in EC will result in altered levels of a myriad of proteins and post‐translationally modified proteins, including phosphoproteins. We recently reported the proteomic and phosphoproteomic effects of knocking in the FBXW7‐R465, ‐R479, or ‐R505 mutations into serous and high‐grade EC cells [10, 11, 12]. Here, we aimed to determine the proteomic and phosphoproteomic effects of *endogenous FBXW7* mutations in EC cells. Using liquid chromatography–tandem mass spectrometry (LC–MS/MS), we profiled the proteomes and phosphoproteomes of two moderately differentiated EC cell lines (HEC‐1‐B^FBXW7−R367X^ and JHUEM‐1^FBXW7*−*R505C^) and isogenic mutation‐corrected cell lines that we previously generated by CRISPR‐Cas9 editing [10, 12]. We identified 98 total proteins at significantly (*p* < 0.05) different levels in both HEC‐1‐B^FBXW7−R367X^ and JHUEM‐1^FBXW7*−*R505C^ cells as compared with the isogenic mutation‐corrected cells. Intriguingly, we found that both of the endogenously mutated EC cell lines had significantly increased levels of TROP2 (Trophoblast Cell Surface Antigen 2), a druggable protein targeted by the antibody‐drug conjugate Sacituzumab Govitecan (IMMU‐132), which is currently being evaluated in a phase II clinical trial for the treatment of persistent or recurrent EC (https://clinicaltrials.gov/study/NCT04251416) (See interim results at https://ascopubs.org/doi/abs/10.1200/JCO.2020.38.15_suppl.6081).

## 2 Results

**FIGURE 1:**
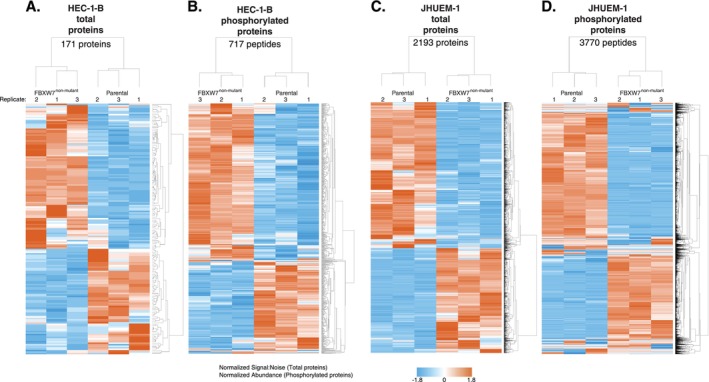
Unsupervised hierarchical clustering of normalized signal‐to‐noise or total proteome peptides exhibiting significantly (*p* < 0.05) different levels in (A) HEC‐1‐B^FBXW7−R367X^ and (C) JHUEM‐1^FBXW7−R505C^ mutant parental cells compared to isogenic mutation‐corrected cells in replicate lysates prepared for each cell line. Unsupervised hierarchical clustering of normalized abundance of filtered phosphorylated peptides exhibiting significantly (*p* < 0.05) different levels in (B) HEC‐1‐B^FBXW7‐R367X^ and (D) JHUEM‐1^FBXW7−R505C^ parental cells compared to isogenic mutation‐corrected cells in replicate lysates prepared for each cell line. Phosphopeptides shown met the following filtering criteria: ≥ ± 2.0 average fold change, maximum abundance > 1,000,000, and coefficient of variance (CV) ≤ 50% for peptides with ≤ 10 average fold change or CV ≤ 80% for peptides with > 10 average fold change. Fold change is not shown on this figure and was calculated within replicates, comparing values in isogenic mutation‐corrected cells to parental cells. For each heatmap, the ordered (top to bottom) protein names are provided in Tables S5–S8 and fold change values are provided in Figures 2 and 3 and Tables S1–S4.

**FIGURE 2:**
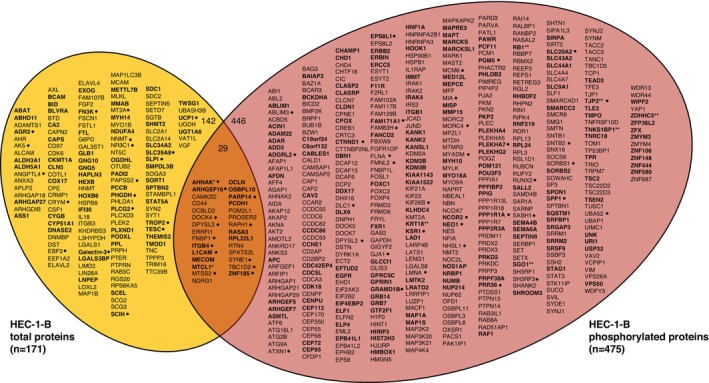
Venn diagram of total (left) and phosphorylated (right) proteins exhibiting significantly (*p* < 0.05) different levels in HEC‐1‐B^FBXW7−R367X^ parental cells compared to isogenic mutation‐corrected cells. Among total proteins with significantly different levels in JHUEM‐1^FBXW7*−*R505C^ cells compared to isogenic mutation‐corrected cells, 17% (29/171) also exhibited significantly different levels of phosphorylation. Proteins that exhibited increased levels in HEC‐1‐B^FBXW7‐R367X^ mutant parental cells compared to isogenic mutation‐corrected cells are indicated in bold. • indicates proteins with *a* > 5‐fold change. *Phosphopeptides for AHNAK and MTCL1 showed significantly increased and decreased levels; total levels of AHNAK and MTCL1 were significantly decreased. **Phosphopeptides for KRT18, RB1, SGO1, TJP2, TNKS1BP1, and ZDHHC5 showed significantly increased and decreased levels. Fold change values and p‐values are provided in Tables S1 and S2.

A comparison of mass spectrometry‐detected protein levels in HEC‐1‐B^FBXW7−R367X^ parental cells to those in isogenic mutation‐corrected cells revealed significantly different (*p* < 0.05) levels of 171 total proteins and 717 phosphorylated peptides (corresponding to 475 unique phosphorylated proteins), all of which were reproducibly altered in triplicate lysate preparations (Figure 1A,B, Tables S1 and S2). Among total proteins with significantly different levels in HEC‐1‐B^FBXW7−R367X^ parental cells compared to isogenic mutation‐corrected cells, 17% (29/171) also exhibited significantly different levels of phosphoproteins; considering total and phosphorylated proteins combined, fold change > 5 was observed in 11% (67/617) of proteins (Figure 2). The significantly altered proteins and phosphoproteins observed between the HEC‐1‐B cell lines included 18% (17/94) of known and predicted FBXW7 substrates [13] (Figure S1), 7% (8/114) of proteins encoded by genes in an expression signature that predicts *FBXW7* mutation status in human cancer [14] (Figure S2), 5% (22/456) of proteins encoded by orthologs of murine genes differentially expressed following re‐introduction of *Fbxw7* into uterine carcinosarcoma cells from *Fbxw7*/*Pten* deficient animals [9] (Figure S3), 3% (30/929) of proteins encoded by ubiquitin‐related genes and 4% (4/95) of proteins encoded by deubiquitinating genes [15] (Figure S4).

**FIGURE 3:**
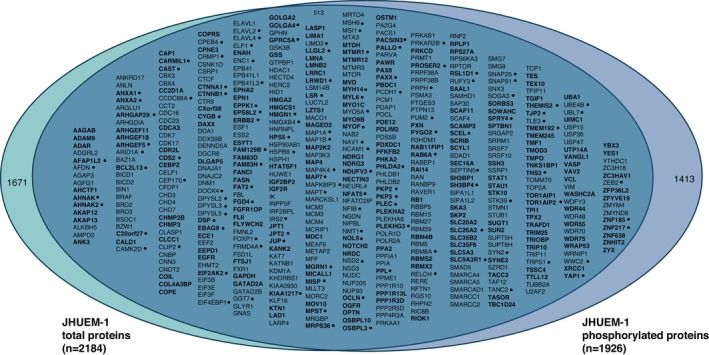
Venn diagram of total (left) and phosphorylated (right) proteins exhibiting significantly (*p* < 0.05) different levels in JHUEM‐1^FBXW7−R505C^ parental cells compared to isogenic mutation‐corrected cells. Among total proteins with significantly different levels in JHUEM‐1^FBXW7−R505C^ cells compared to isogenic mutation‐corrected cells, 23% (513/2184) also exhibited significantly different levels of phosphorylation. Proteins that exhibited increased levels in JHUEM‐1^FBXW7‐R505C^ mutant cells compared to isogenic mutation‐corrected cells are in bold. • indicates total proteins with *a* > 5‐fold change. Fold change values are provided in Tables S3 and S4.

Comparing JHUEM‐1^FBXW7−R505C^ cells to the isogenic mutation‐corrected cells, we found 2193 total proteins (including 2 isoforms for 9 proteins) and 3770 phosphorylated peptides (corresponding to 1926 unique phosphorylated proteins) that exhibited significantly different levels, all of which were reproducibly altered in triplicate lysate preparations (Figure 1C,D, and Tables S3 and S4). Among the total proteins with significantly different levels in JHUEM‐1^FBXW7−R505C^ cells compared to the isogenic mutation‐corrected cells, 23% (513/2184) also exhibited significantly different levels of phosphorylation; among these proteins, 25.7% (132/513) have a fold change > 5 for the total protein level (Figure 3). Proteins and/or phosphoproteins present at significantly different levels between the JHUEM‐1 cell lines included 57% (54/94) of known and predicted FBXW7 substrates [13] (Figure S1), and proteins corresponding to 40% (46/114) of genes in an expression signature that predicts *FBXW7* mutation status [14] (Figure S2), 13% (61/456) of proteins encoded by orthologues of murine genes differentially expressed following re‐introduction of *Fbxw7* into uterine carcinosarcoma cells from *Fbxw7*/*Pten* deficient animals [9] (Figure S3), 25% (230/929) of ubiquitin‐related genes and 24% (23/95) of deubiquitinating genes [15] (Figure S4).

**FIGURE 4:**
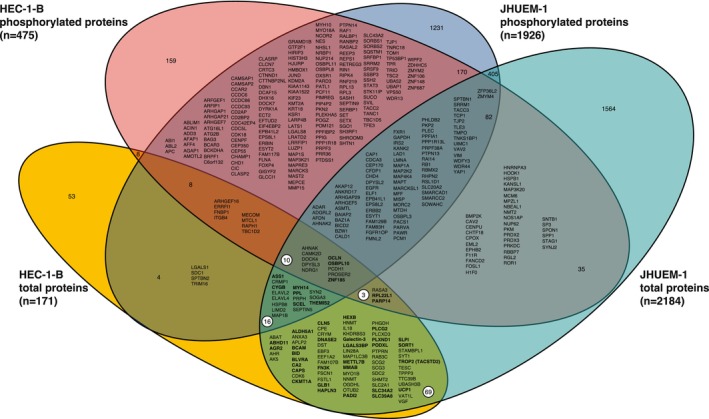
Venn diagram of total and phosphorylated proteins exhibiting significantly (*p* < 0.05) different levels in HEC‐1‐B^FBXW7−R367X^ and JHUEM‐1^FBXW7−R505C^ parental cells compared to isogenic mutation‐corrected cells. White circles indicate the 98 “total proteins” detected at significantly different total protein levels in both HEC‐1‐B and JHUEM‐1, including 40 proteins (bold) at higher levels in *FBXW7* mutated cells compared to isogenic *FBXW7* mutation‐corrected cells.

We identified 397 phosphorylated and/or total proteins that exhibited significantly different levels in the *FBXW7*‐mutant parental cell line compared to the isogenic mutation‐corrected cell line for both HEC‐1‐B and JHUEM‐1 (Figure 4). Ninety‐eight proteins exhibited significantly different *total* protein levels in both HEC‐1‐B^FBXW7−R367X^ and JHUEM‐1^FBXW7*−*R505C^, 40 of which exhibited higher levels in the *FBXW7*‐mutant parental lines compared to the isogenic mutation‐corrected lines (Figure 4); 40 of the remaining 58 proteins exhibited lower levels in the *FBXW7*‐mutant parental lines compared to the isogenic CRISPR‐edited *FBXW7* non‐mutant derivative lines. A majority (22/40) of the proteins exhibiting higher levels in *FBXW7*‐mutant cells annotated to the gene ontology (GO) term *vesicle* (GO:0031982); the most significantly associated gene ontology terms were *extracellular exosome* (GO:0070062), *extracellular vesicle* (GO:1903561) and *extracellular organelle* (GO:0043230), which were all comprised of the same set of 18 proteins (ASS1, BCAM, BLVRA, CA2, CLN5, DNASE2, GLB1, HEXB, galectin‐3 (*LGALS3*), LGALS3BP, MYH14, PADI2, PLCG2, PODXL, PPL, SCEL, SLPI, TROP2 (*TACSTD2*)) with significantly different levels in *FBXW7*‐mutant HEC‐1‐B^FBXW7−R367X^ and JHUEM‐1^FBXW7−R505C^ cells compared to the isogenic mutation‐corrected cell lines (Table S9). The gene ontology terms most significantly associated with proteins that exhibited lower levels in *FBXW7*‐mutant cells were *cytoskeletal protein binding* (GO:0008092), *protein localization to secretory granule* (GO:0033366), *transport vesicle* (GO:0030133), and *transport vesicle membrane* (GO:0030658) (Table S10). GO terms that were most significantly associated with the 98 total proteins present at significantly different levels in both *FBXW*7‐mutant cell lines were *identical protein binding* (GO:0042802), *gamma‐aminobutyric acid metabolic process* (GO:0009448), *neurotransmitter catabolic process* (GO:0042135), and *gamma‐aminobutyric acid catabolic process* (GO:0009450) (Table S11).

**FIGURE 5:**
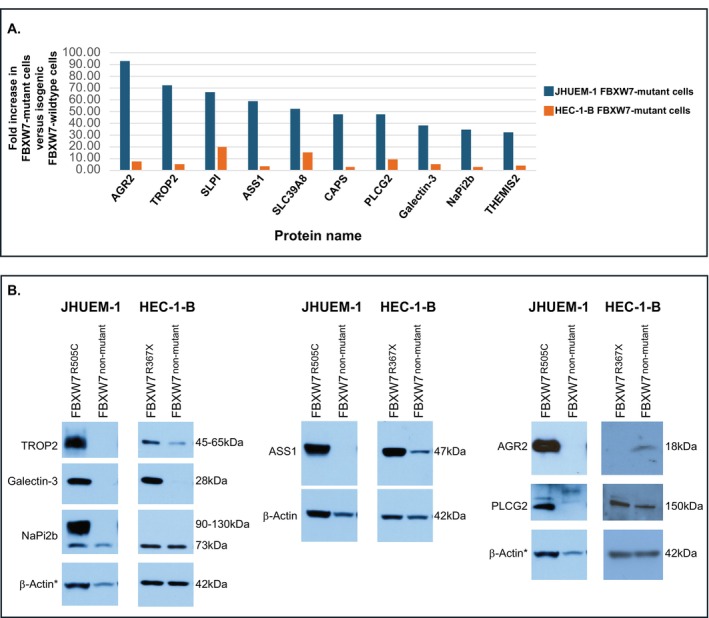
(A) Total proteins that exhibited significantly higher mass spectrometry‐detected protein levels in both HEC‐1‐B^FBXW7−R367X^ and JHUEM‐1^FBXW7−R505C^ mutant lines, compared to the isogenic mutation‐corrected cells, and exhibited a normalized fold change of ≥ 30‐fold in JHUEM‐1^FBXW7−R505C^ cells. Bars represent average fold change in JHUEM‐1 (blue) or HEC‐1‐B (orange) *FBXW7‐*mutant parental cells compared to isogenic mutation‐corrected cells. (B) Western blot detection of six proteins‐of‐interest in JHUEM‐1 and HEC‐1‐B parental FBXW7‐mutant cells compared to isogenic mutation‐corrected cells. *, the images for β‐Actin loading controls for JHUEM‐1 TROP2/galectin‐3/NaPi2b (left panel) and AGR2/PLCG2 (right panel) are the same because these proteins were detected on a single membrane (Figure S5). Uncropped images of all Western blots are provided in Figures S5 and S6.

Ten significantly altered total proteins that increased in both HEC‐1‐B^FBXW7−R367X^ and JHUEM‐1^FBXW7−R505C^ mutant lines, compared to the isogenic mutation‐corrected cells, exhibited a normalized fold change of ≥ 30‐fold in JHUEM‐1^FBXW7−R505C^ cells (Figure 5A). We used Western blotting to orthogonally assess levels of six of these proteins (TROP2 (*TACSTD2*), galectin‐3 (*LGALS3*), ASS1, PLCG2, AGR2, NaPi2b (*SLC34A2*)) for which antibodies were available from Cell Signaling Technology. Possibly due to the small fold change observed for AGR2 and NaPi2b (*SLC34A2*) in HEC‐1‐B^FBXW7‐R367X^ (Figure 5A), and the differential sensitivities of LC–MS/MS and Western blotting, we did not observe increased levels of AGR2 (Anterior gradient protein 2 homolog) or NaPi2b (Sodium‐dependent phosphate transport protein 2B) in HEC‐1‐B^FBXW7‐R367X^ cells, compared to isogenic mutation‐corrected cells (Figure S6). We confirmed increased levels of the remaining four proteins, namely TROP2 (Trophoblast cell surface antigen 2), galectin‐3, ASS1 (Argininosuccinate synthase 1) and PLCG2 (Phospholipase C gamma 2), in HEC‐1‐B^FBXW7−R367X^ cells and of all six proteins in JHUEM‐1^FBXW7‐R505C^ cells, compared to isogenic mutation‐corrected lines (Figure 5B, Figures S5 and S6).

## 3 Materials and Methods

### 3.1 Ethics Statement

The research conducted in this study was excluded from IRB Review per the Common Rule 45 CFR 46 and NIH policy for the use of specimens/data.

### 3.2 Cell Culture

HEC‐1‐B was originally established from a moderately differentiated papillary adenocarcinoma [16]. JHUEM‐1 was established from a grade‐2 EEC (URL: https://cellbank.brc.riken.jp/cell_bank/CellInfo/?cellNo=RCB1548). HEC‐1‐B EC cells were acquired from ATCC (HTB‐113) and maintained in EMEM + 10% FBS at 37°C in a humidified atmosphere with 5% CO_2_. JHUEM‐1 EEC cells were purchased from Riken Bioresource Research Center and maintained in DMEM/HamF12 + 15% FBS at 37°C in a humidified atmosphere with 5% CO_2_. Cells were authenticated by Laragen Inc. (Culver City, CA) using short tandem repeat profiling prior to shipment to the Washington University Genome Engineering and iPSC Center (GEIC; St. Louis, MO). All cell lines were verified mycoplasma free by the Washington University GEIC and re‐authenticated by Laragen Inc. (Culver City, CA) at the time frozen stocks were established. Both cell lines were previously inferred to harbor microsatellite instability [17]; HEC‐1‐B was reported to have an *FBXW7* deep deletion, while JHUEM‐1 had no detectable *FBXW7* copy number aberration [17].

### 3.3 Generation of CRISPR‐Edited FBXW7 Non‐Mutated Cell Lines

HEC‐1‐B and JHUEM‐1 cells were CRISPR‐edited by the GEIC at Washington University, St. Louis, to correct the endogenous *FBXW7* c.C1099T (p.R367X) and c.C1513T (p.R505C) mutations, respectively, following methods published previously [11].

### 3.4 DNA Extraction, Polymerase Chain Reaction (PCR) Amplification and Sanger Sequencing

DNA was extracted using the Gentra Puregene Kit (Qiagen, Germantown, MD) according to the manufacturer's instructions. Verification of single mutations and wildtype status of all remaining coding exons of *FBXW7* in JHUEM‐1 and HEC‐1‐B parental cells and wildtype status of all *FBXW7* coding exons of CRISPR‐edited derivative lines (Figure S1) was verified using *FBXW7* primers [18], PCR conditions, purification, and Sanger sequencing exactly as previously reported [10].

### 3.5 Lysate Preparation and Liquid Chromatography Tandem Mass Spectrometry (LC–MS/MS) Analysis

HEC‐1‐B^FBXW7−R367X^ (passage (P)5), HEC‐1‐B mutation corrected (P5), JHUEM‐1^FBXW7−R505C^ (P3), and JHUEM‐1 mutation corrected (P5) cells were counted with a Countess Cell Counter (Thermo Fisher Scientific, Waltham, MA), plated into four 150 mm dishes at a density of 4 × 10^6^ cells per dish, and scraped into a total of 4 mL urea lysis buffer (Cell Signaling Technology (CST), Danvers, MA) the following day. Triplicate lysates were prepared for each cell line from the same passage number to minimize proteomic variation due to cell age and were immediately frozen on dry ice before shipment to CST. Sonication, centrifugation, reduction, digestion, and purification of cellular extracts and LC–MS/MS analysis were performed exactly as previously described [11]. Also, as previously described [11], Immobilized Metal Affinity Chromatography (IMAC) enrichment with Fe‐NTA magnetic beads (CST) was used for phosphorylated protein analysis, and labeling with TMT10plex reagent (Thermo Fisher Scientific) was used for total protein analysis. LC–MS/MS analysis was performed using an Orbitrap‐Fusion Lumos Tribrid mass spectrometer (Thermo Fisher Scientific) with replicate injections of each sample for the IMAC analysis. An HCD‐MS2 acquisition method was used for phosphopeptide analysis, and an MS3 acquisition method was used for total proteome analysis. Details of acquisition methods have been previously provided [11].

### 3.6 Peptide and Protein Identification

Mass spectra evaluations and peptide and protein identification were performed by CST exactly as previously described [11].

### 3.7 Filtering to Proteins With Significantly Different Levels in FBXW7‐Mutated Parental Cells

Proteins exhibiting significantly different levels in *FBXW7* non‐mutant CRISPR‐edited HEC‐1‐B and JHUEM‐1 cells compared to isogenic *FBXW7*‐mutant parental cells were determined using a previously published filtering scheme [11]. Briefly, total proteins with ≥ ± 2.0 average fold change were filtered to those with significantly (*p* < 0.05) different levels in *FBXW7* non‐mutant CRISPR‐edited cells compared to isogenic *FBXW7*‐mutant parental cells. *p*‐values were based on a two‐tailed *t*‐test using the signal:noise for each CRISPR‐edited derivative line versus isogenic parental cells across three replicates. Phosphorylated peptides were filtered using these same criteria with additional filters of maximum abundance > 1,000,000 and percent coefficient of variance (%CV) ≤ 50% for peptides with ≤ 10 average fold change or ≤ 80% for peptides with > 10 average fold change. Unsupervised hierarchical clustering was performed using Partek Genomics Suite.

### 3.8 Functional Annotation

Proteins were functionally annotated using g:Profiler [19] (RRID :S CR_008653) and Ingenuity Pathway Analysis (IPA) (QIAGEN Inc., https://www.qiagenbioinformatics.com/products/ingenuitypathway‐analysis).

### 3.9 Western Blot Analysis

For HEC‐1‐B^FBXW7−R367X^ (P8), HEC‐1‐B mutation corrected (P7 or P8), JHUEM‐1^FBXW7−R505C^ (P11), and JHUEM‐1 mutation corrected (P11) cell lines, protein isolation, quantification, gel electrophoresis, and transfer were performed as previously described [10, 12], from cells collected 1 day after plating 1.6 × 10^6^ cells per 100 mm dish, in RIPA buffer (Thermo Fisher Scientific) containing 1× Protease/Phosphatase inhibitor cocktail (#5872; Cell Signaling Technology). Triplicate lysates were prepared for each cell line from the same passage number to minimize proteomic variation due to cell age.

Proteins were subjected to electrophoresis into 10% Mini‐PROTEAN‐TGX gels or 4%–15% gradient Mini‐PROTEAN‐TGX gels (Bio‐Rad Laboratories) and wet transferred to PVDF membranes (Bio‐Rad Laboratories, Hercules, CA). Blots were blocked in tris‐buffered saline containing 0.1% Tween 20 (TBST) + 5% milk and incubated in the following antibodies from Cell Signaling Technology and R&D systems according to their suggested protocols at dilutions of 1:1000: AGR2 (#13062), LGALS3 (#87985), TACSTD2/TROP2 (#47866), PLCG2 (#3872), NaPi2b/SLC34A2 (#66445), ASS1 (#70720). As a loading control, blots were incubated in β‐Actin antibody (A2228; Sigma‐Aldrich, St. Louis MO) overnight at 4°C at a dilution of 1:10,000 in TBST + 5% BSA. Secondary antibodies were Cell Signaling Technology # 7074 and #7076 used at 1:2000 in TBST + 5% milk and incubated for 1–2 h. Proteins were detected using Clarity and Clarity Max Western ECL Substrate (Bio‐Rad Laboratories) prior to film exposure (Carestream Health Inc., Rochester, NY or Agfa Healthcare NV, Mortsel, Belgium) and development using a film processor (Konica Minolta, Ramsey, NJ). To enable probing with multiple antibodies, blots were incubated in Restore Western Blot Stripping Buffer (Thermo Fisher Scientific). All Western blots are representative of a minimum of three lysate replicates.

## 4 Discussion

In the present study, we searched for proteomic and phosphoproteomic changes associated with endogenous *FBXW7* mutations in the moderately differentiated EC cell lines, HEC‐1‐B^FBXW7*−*R367X^ and JHUEM‐1^FBXW7*−*R505C^. To more accurately reflect tumor cells in vivo, we utilized unsynchronized cell lines for these experiments. It is conceivable that synchronized cell populations might yield different results for proteins that are present at variable levels throughout the cell cycle. In our experimental conditions, we identified 3809 proteins and/or phosphoproteins that exhibited increased or decreased levels in at least one of the mutated cell lines compared to isogenic mutation‐corrected cells; the levels of 98 total proteins and 270 phosphoproteins were altered in both mutant cell lines. The 98 total proteins included TROP2 (*TACSTD2*), a protein being clinically evaluated as a druggable target for EC, and galectin‐3 (*LGALS3*), which is a potentially druggable target.

TROP2 is a transmembrane glycoprotein that regulates cell proliferation, migration, and intracellular signal transduction pathways (reviewed in [20]). Upregulation of this protein has been detected by IHC in 60% of SECs and 84% of EECs [21, 22]. Among endometrioid tumors, strong expression of TROP2 was associated with higher tumor grade and cervical involvement, which are adverse prognosticators [23, 24]. The mechanistic basis for increased TROP2 protein levels in *FBXW7*‐mutated EC cells remains to be determined. One possible mechanism is that the FBXW7‐SKP1‐CUL1 ubiquitin ligase complex regulates TROP2 via ubiquitin‐mediated degradation, and therefore mutations disrupting the FBXW7 WD repeats result in the loss of FBXW7/TROP2 binding and elevated TROP2 levels. Alternatively, FBXW7 substrates, or their downstream effectors, might regulate TROP2 levels either transcriptionally or post‐translationally. In this regard, it is interesting to speculate that galectin‐3 might mediate the effect of FBXW7 on TROP2 levels because galectin‐3 positively regulates *TROP2* (*TACSTD2*) gene expression in colorectal cancer cells [25] and both galectin‐3 and TROP2 exhibited increased levels in the two *FBXW7*‐mutated EC cell lines in our study.

Galectin‐3 is a β‐galactosidase binding protein involved in the regulation of apoptosis, cell cycle, cell growth, metastasis, and other cellular processes [26]. Our study is the first to report that increased galectin‐3 protein levels are associated with *FBXW7* mutations in EC cells. Dysregulation of this protein has previously been reported in EC, but results were conflicting. Whereas some studies observed galectin‐3 upregulation in EC as compared with control tissues [27], others observed downregulation [28, 29, 30, 31] or no change in expression [32]. It has been suggested that these contradictory findings might be due to methodological variability across studies [27]. It is also possible that the analysis of unsynchronized cell populations may contribute to the interstudy variability regarding galectin‐3 levels in EC cells. Our results raise the additional possibility that interstudy differences in the *FBXW7* mutation status of endometrial tumors may be an additional confounding factor contributing to these contradictory findings.

Interestingly, several of the orthogonally validated proteins that exhibited increased levels in one or both cell lines with endogenous *FBXW7* mutations in our study are druggable or potentially druggable [33, 34, 35], including galectin‐3 ([36]; reviewed in [37]) and TROP2 [38]. A recent preclinical study using serous EC cell lines, xenografts, and patient‐derived organoids showed that galectin‐3 knockout or treatment with a galectin‐3 inhibitor resulted in the reduction of several tumorigenic features, including reduced tumor growth in vivo [36]. Regarding druggability, TROP2 is particularly interesting because sacituzumab govitecan (SG) (an antibody‐drug conjugate that targets TROP2 and which has SN‐38, an active metabolite of irinotecan, as the payload) is currently being evaluated for the treatment of EC. Preclinical studies in EC cells have shown that increased TROP2 levels are a biomarker of sensitivity to killing by SG. Moreover, SG exhibited antitumor activity in TROP2‐positive EC cell line‐derived xenografts [21, 22]. Clinical trials evaluating SG for the treatment of EC have thus far yielded encouraging results [39, 40, 41] (ClinicalTrials.gov Identifier: NCT04251416) (URL: https://classic.clinicaltrials.gov/ct2/show/NCT04251416) [40]. Given our finding that endogenous *FBXW7* mutations are associated with increased levels of TROP2 in EC cells, it would be interesting, in future studies, to determine whether *FBXW7* mutation status, either alone or in combination with TROP2 protein status, is a molecular correlate of clinical responsiveness of EC patients to SG.

The clinical management of ECs is continuing to evolve with increased knowledge of the molecular landscapes of these tumors. Surrogate tests for the four TCGA prognostic subgroups, based on determining *POLE*, MSI, and p53 status, have been developed and validated ([42, 43]; and reviewed in [4, 44, 45, 46]). The FIGO staging system for EC was recently revised to include consideration of additional features including molecular subtype based on the results of surrogate testing [47], although this has been somewhat controversial [48, 49, 50]. It should be noted, however, that although advances have been made in disease risk stratification over the past decade, there are still some challenges to incorporating molecular subtyping of EC into routine clinical practice (reviewed in [46]). Despite this, a better understanding of the molecular and proteomic landscapes of ECs provides momentum for useful clinical developments that could benefit EC patients. For example, we previously reported that FBXW7‐mutant EC cells exhibit significantly altered levels of L1CAM as compared to their isogenic FBXW7‐wildtype counterparts [12]. L1CAM is being studied for its value as a prognostic biomarker, and overexpression has recently been associated with adverse outcomes [43, 51, 52, 53, 54, 55, 56, 57].

In conclusion, our study provides novel insights into the proteomic and phosphoproteomic effects of endogenous *FBXW7* mutations in EC cells, including thousands of significantly altered protein levels as well as increased levels of galectin‐3 and the druggable target TROP2. Future studies to (1) elucidate the mechanisms underlying increased levels of these proteins in *FBXW7*‐mutant cell lines, (2) determine whether the increased levels of TROP2 and galectin‐3 associated with *FBXW7* mutations are detectable on EC tissue specimens by immunohistochemistry and whether the combined results are prognostic and/or predictive, and (3) determine whether *FBXW7* mutation status is a biomarker of clinical responsiveness to SG are warranted.

## Author Contributions

**Mary Ellen Urick:** conceptualization (equal); project administration (equal); investigation (lead); formal analysis (lead); writing‐original draft (equal); writing – review and editing (equal); visualization (lead). **Suresh Kumar Chalapareddy:** investigation (equal); writing – review and editing (equal); visualization (equal). **Eun‐Jeong Yu:** investigation (equal); writing – review and editing (equal); visualization (equal). **Daphne W. Bell:** conceptualization (equal); funding acquisition (lead); project administration (equal); supervision (lead); writing – original draft (equal); writing – review and editing (equal); visualization (equal).

## Ethics Statement

The research conducted in this study was excluded from IRB Review per the Common Rule 45 CFR 46 and NIH policy for the use of specimens/data.

## Conflicts of Interest

Dr. Mary Ellen Urick, Dr. Suresh Kumar Chalapareddy, and Dr. Eun‐Jeong Yu have no conflicts of interest. Dr. Daphne W. Bell is an inventor on US patent no. 7,294,468, which has been licensed and provides royalty income for work performed outside the current study.

## Supporting information


Figure S1.

Table S1.

## Data Availability

The data that support the findings of this study will be available from the corresponding author upon reasonable request.
