# Supplementary material for: Proteomic Changes Associated With Endogenous FBXW7 Mutations in Moderately Differentiated Endometrial Cancer Cells Include Increased TROP2 and Galectin‐3 Levels
Source: Cancer Med. 2025 Mar 14;14(6):e70765. doi: 10.1002/cam4.70765 (PMC11909011; doi:10.1002/cam4.70765)
Supplement: Supplementary file 1 — Figure S1. [file CAM4-14-e70765-s001.pdf]

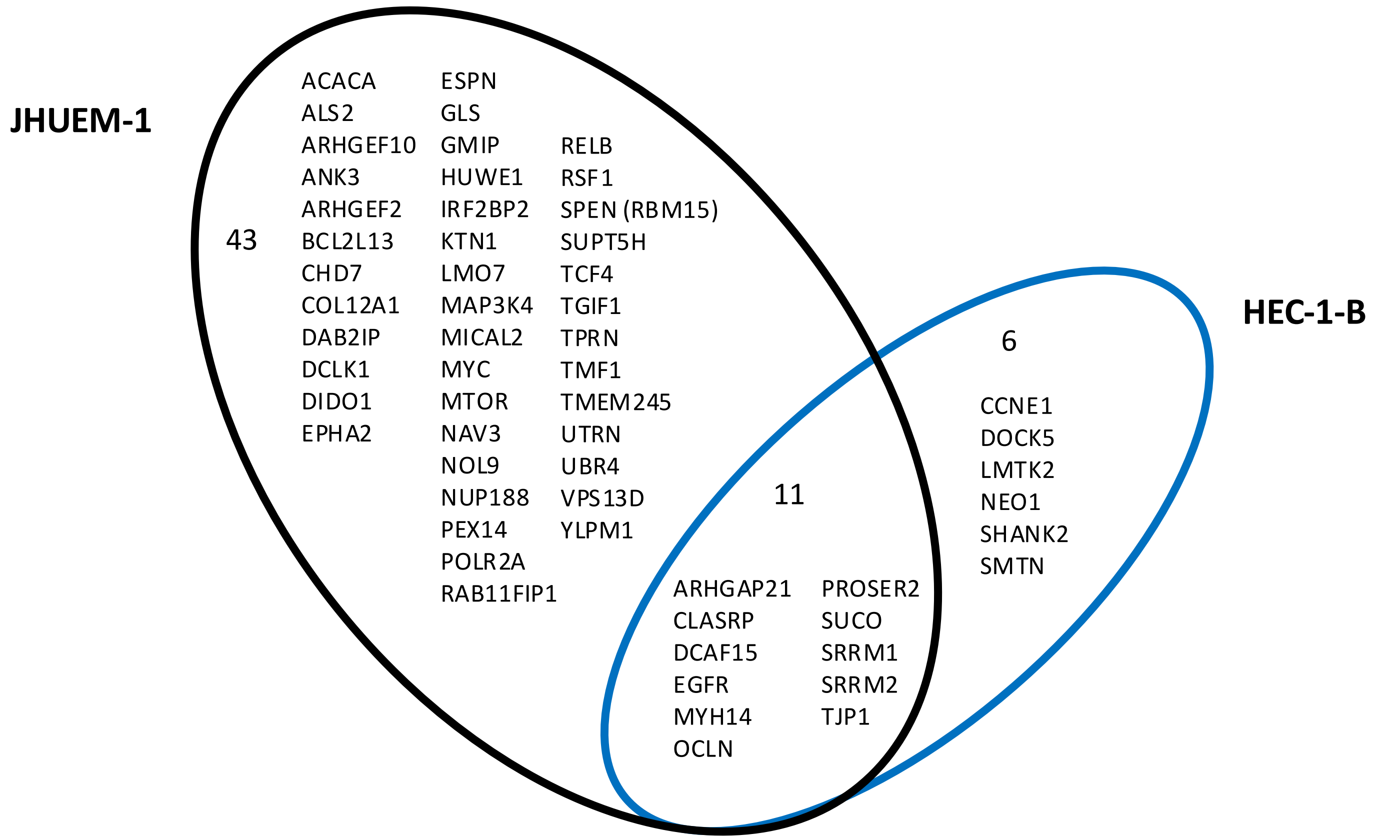

Supplemental Figure S1. Venn diagram of total and/or phosphorylated proteins exhibiting significantly (*P* < .05) different levels (≥+/- 2.0 average fold change) in HEC-1-B$^{FBXW7\text{-}R367X}$ and JHUEM-1$^{FBXW7\text{-}R505C}$ parental cells compared to isogenic mutation-corrected cell lines that are included in a published list of 94 known/predicted FBXW7 substrates (Arabi et al., 2012).

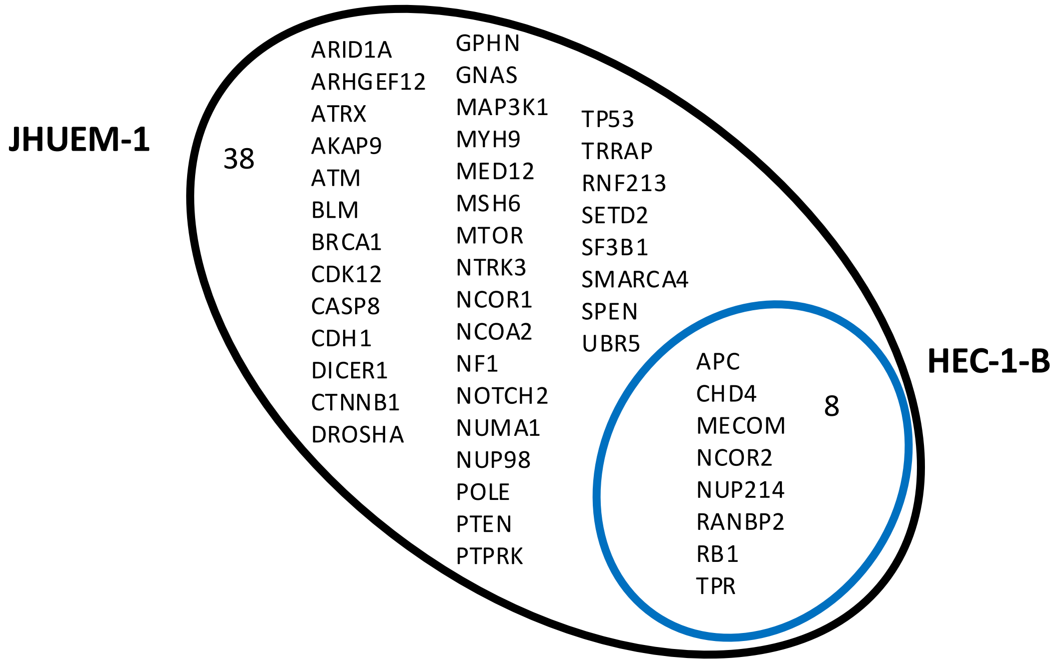

Supplemental Figure S2. Venn diagram of total and/or phosphorylated proteins exhibiting significantly ($P < 0.05$) different levels (≥+/- 2.0 average fold change) in HEC-1-B$^{FBXW7\text{-}R367X}$ and JHUEM-1$^{FBXW7\text{-}R505C}$ parental cells compared to isogenic mutation-corrected cell lines and that are included in a 114 gene pan-cancer transcriptional signature implicating FBXW7 in cancer cell oxidative metabolism (Davis et al., 2018).

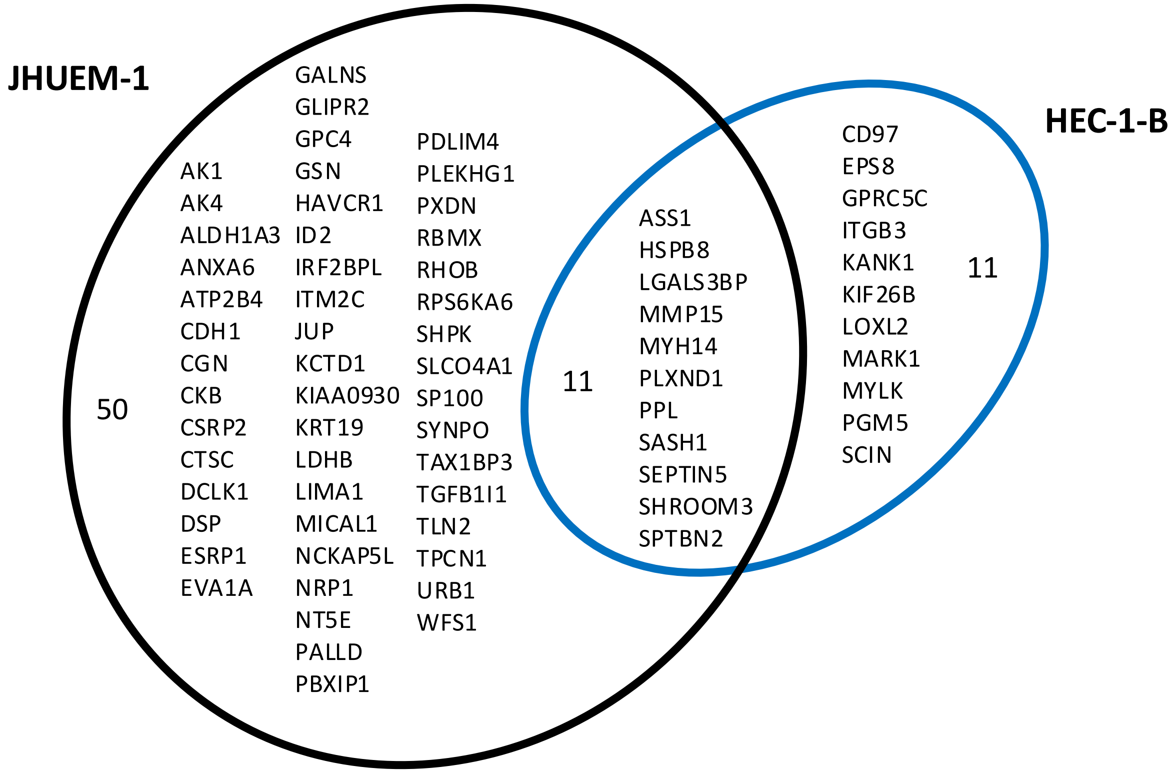

Supplemental Figure S3. Venn diagram of total and/or phosphorylated proteins exhibiting significantly (*P* < 0.05) different levels (≥+/- 2.0 average fold change) in HEC-1-B$^{\text{FBXW7-R367X}}$ and JHUEM-1$^{\text{FBXW7-R505C}}$ parental cells compared to isogenic mutation-corrected cell lines and that are orthologues of murine genes differentially expressed following re-introduction of *Fbxw7* into uterine carcinosarcoma cells from *Fbxw7/Pten* deficient animals (Cuevas et al., 2019).

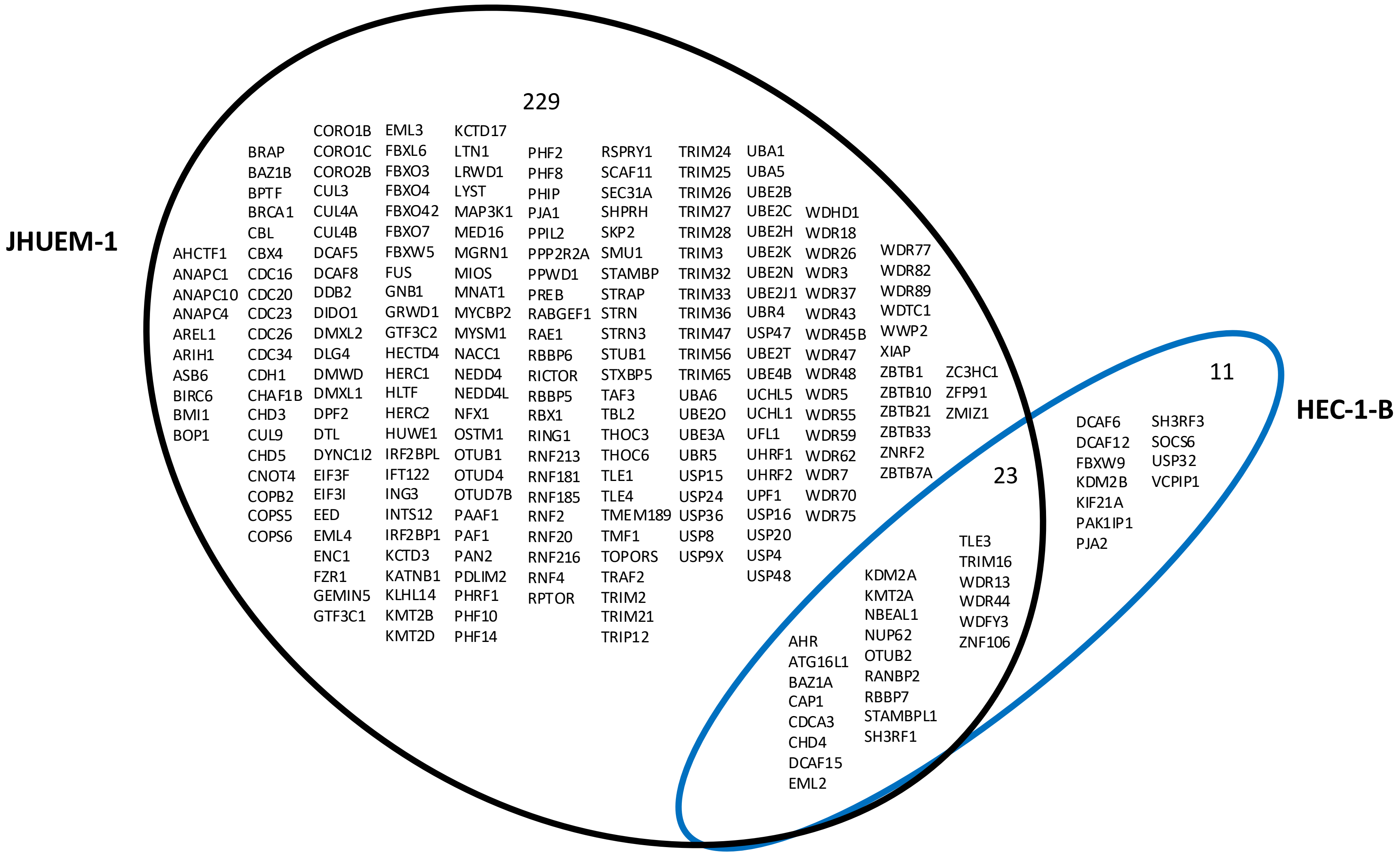

Supplemental Figure S4. Venn diagram of total and/or phosphorylated proteins exhibiting significantly ($P < 0.05$) different levels (≥+/- 2.0 average fold change) in HEC-1-B$^{FBXW7\text{-}R367X}$ and JHUEM-1$^{FBXW7\text{-}R505C}$ parental cells compared to isogenic mutation-corrected cell lines and that are part of a curated list of 1024 ubiquitin-related genes/deubiquitin genes (Ge et al., *Cell Rep*. 2018).

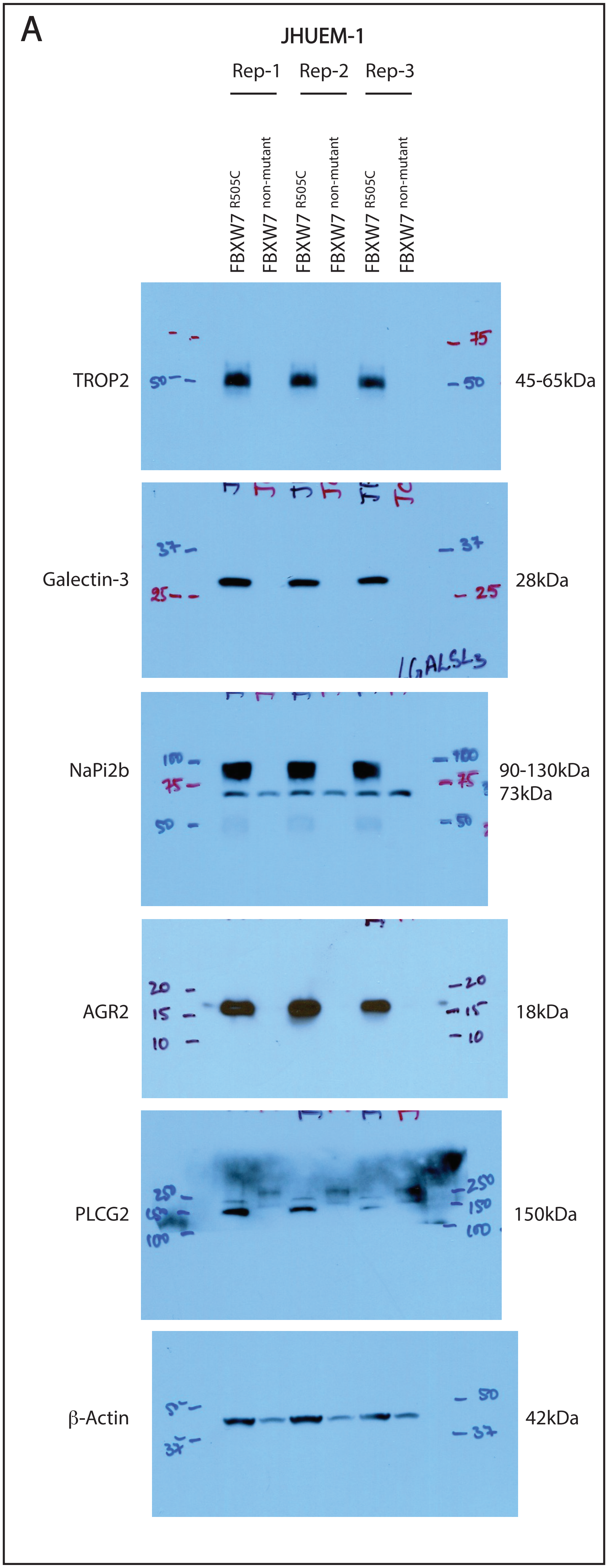

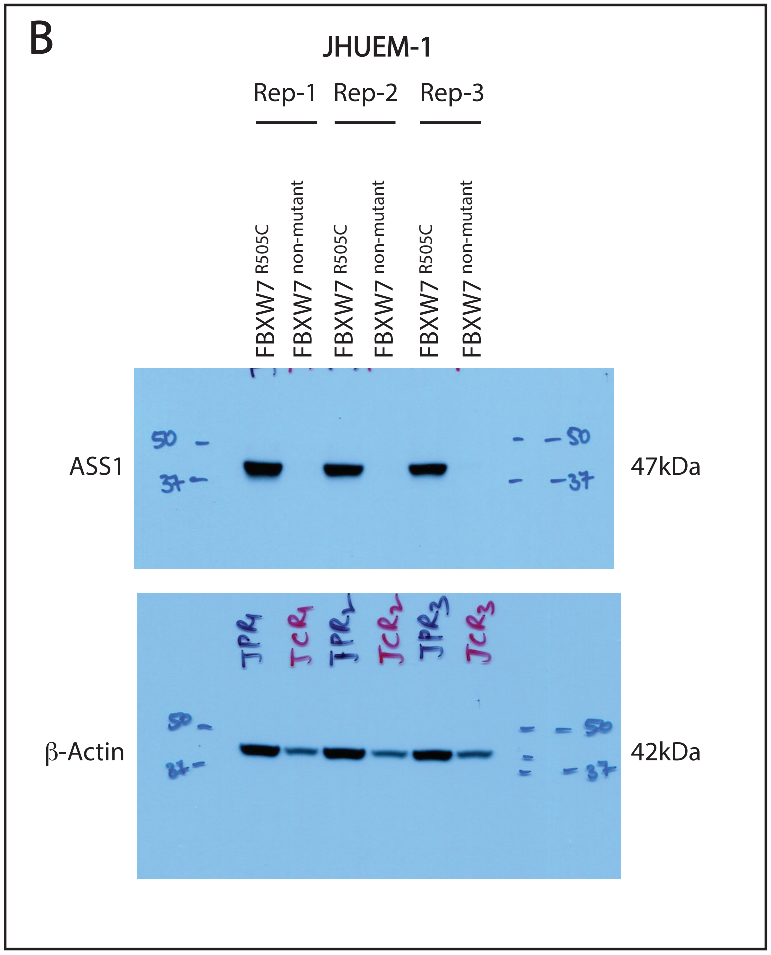

Supplemental Figure S5. Uncropped western blot images for isogenic *FBXW7*-R505C-mutant and mutation-corrected (non-mutant) JHUEM-1 cells. **(A)** and **(B)** represent two different membranes probed for the indicated proteins. Results for three biologic replicates (Reps1-3) are shown. Cropped images of Rep-1 results are displayed in Figure 5B.

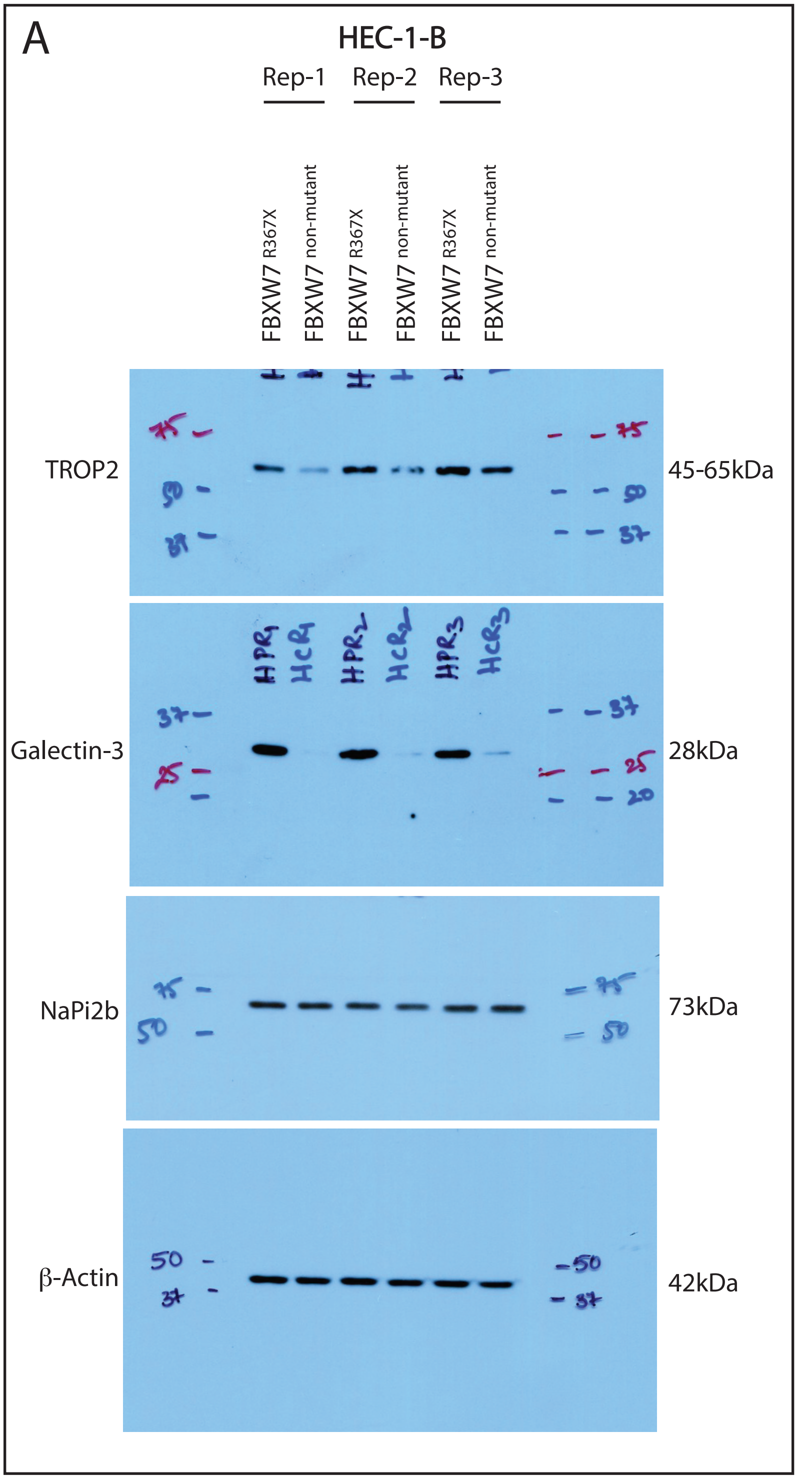

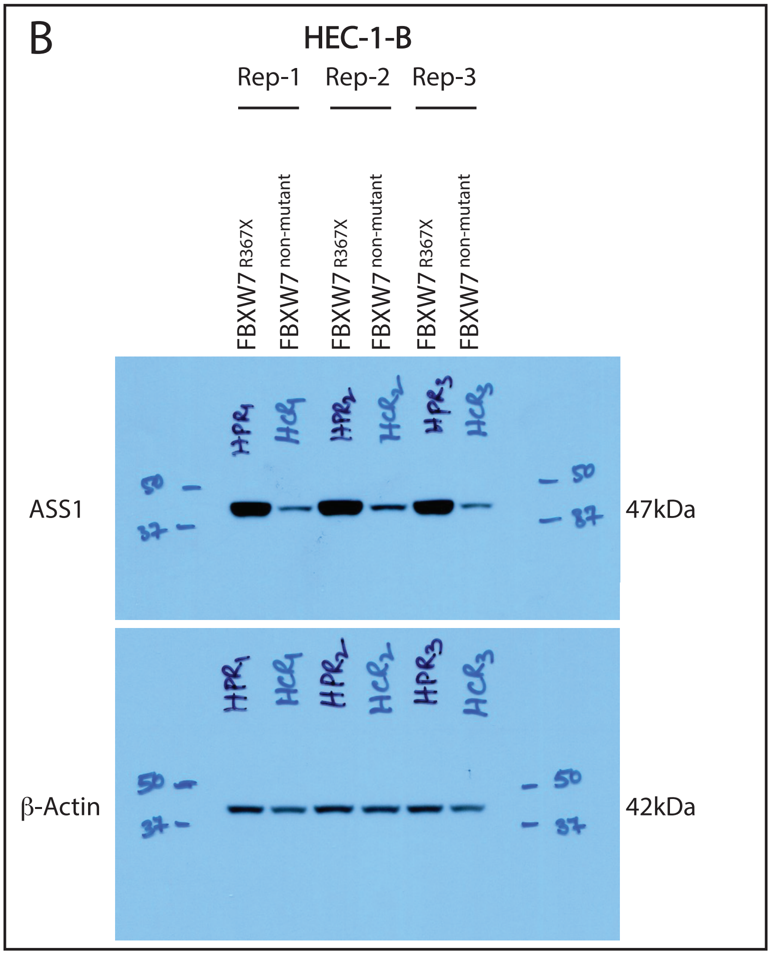

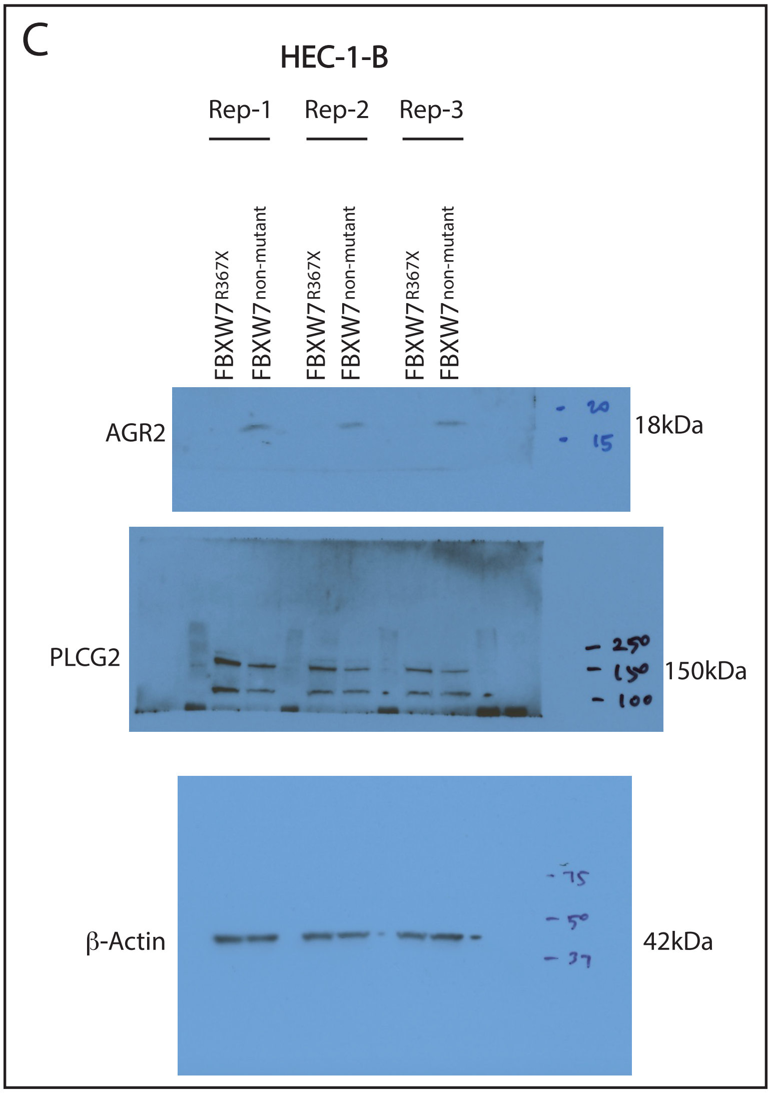

Supplemental Figure S6. Uncropped western blot images for isogenic *FBXW7*-R367X-mutant and mutation-corrected (non-mutant) HEC-1-B cells. **(A)**, **(B)**, and **(C)** represent three different membranes probed for the indicated proteins. Results for three biologic replicates (Reps1-3) are shown. Cropped images of Rep-1 results are displayed in Figure 5B.
